# Supplementary material for: Traumatic Brain Injury in Cameroon: A Prospective Observational Study in a Level I Trauma Centre
Source: Medicina (Kaunas). 2023 Aug 28;59(9):1558. doi: 10.3390/medicina59091558 (PMC10535664; doi:10.3390/medicina59091558)
Supplement: Supplementary file 1 [file medicina-59-01558-s001.zip › medicina-2567465-supplementary.pdf]

Supplementary Table S1: Correlation between time difference upon arrival at the referral hospital and outcome

Characteristic	Overall, N = 160[1]	(<1h), N = 39[1]	[1-4)h, N = 71[1]	(4.1-11.9)h, N = 30[1]	(12-24)h, N = 20[1]	p-value
**Recovery status (DRS) at discharge**						**0.09**
Extreme vegetative state	5 (3.7%)	3 (9.1%)	1 (1.6%)	1 (3.8%)	0 (0%)	
vegetative state	3 (2.2%)	1 (3.0%)	1 (1.6%)	1 (3.8%)	0 (0%)	
Extremely severe disability	6 (4.5%)	3 (9.1%)	1 (1.6%)	1 (3.8%)	1 (7.1%)	
Severe disability	8 (6.0%)	1 (3.0%)	4 (6.6%)	0 (0%)	3 (21%)	
Moderately severe	25 (19%)	4 (12%)	14 (23%)	5 (19%)	2 (14%)	
Moderate	28 (21%)	3 (9.1%)	16 (26%)	7 (27%)	2 (14%)	
Partial disability	26 (19%)	7 (21%)	14 (23%)	5 (19%)	0 (0%)	
Mild disability	8 (6.0%)	4 (12%)	2 (3.3%)	0 (0%)	2 (14%)	
Recovering	25 (19%)	7 (21%)	8 (13%)	6 (23%)	4 (29%)	
**QoLIBRI**						**0.847**
Impaired	34 (27%)	10 (32%)	13 (22%)	6 (24%)	5 (45%)	
Borderline	14 (11%)	3 (9.7%)	8 (13%)	2 (8.0%)	1 (9.1%)	
Normal	33 (26%)	8 (26%)	18 (30%)	5 (20%)	2 (18%)	
Above average	46 (36%)	10 (32%)	21 (35%)	12 (48%)	3 (27%)	
**GOSE**						**0.577**
Death	22 (14%)	5 (14%)	6 (9.0%)	4 (14%)	7 (35%)	
GR-	15 (9.9%)	4 (11%)	8 (12%)	3 (10%)	0 (0%)	
GR+	44 (29%)	9 (25%)	21 (31%)	9 (31%)	5 (25%)	
MD-	23 (15%)	7 (19%)	9 (13%)	4 (14%)	3 (15%)	

MD+	32 (21%)	5 (14%)	17 (25%)	6 (21%)	4 (20%)	
SD-	4 (2.6%)	1 (2.8%)	1 (1.5%)	1 (3.4%)	1 (5.0%)	
SD+	12 (7.9%)	5 (14%)	5 (7.5%)	2 (6.9%)	0 (0%)	
[1]n (%)						

***GOSE:** Glasgow Outcome Scale Extended, **QoLIBRI:** Quality of Life After Brain Injury, **GR-:** Lower good recovery**, GR+:** Upper good recovery, **MD-:** Lower moderate disability, **MD+:** Upper moderate disability, **SD-:** Lower severe disability, **SD+:** Upper severe disability.

Supplementary Table S2: Correlation between direct referral and non-direct referral and outcome

Characteristic	Overall, N = 160[1]	Indirect, N = 95[1]	Direct, N = 65[1]	p-value[2]
**Recovery status (at discharge)**				**0.061**
Extreme vegetative state	5 (3.7%)	1 (1.3%)	4 (7.0%)	
vegetative state	3 (2.2%)	2 (2.6%)	1 (1.8%)	
Extremely severe disability	6 (4.5%)	2 (2.6%)	4 (7.0%)	
Severe disability	8 (6.0%)	6 (7.8%)	2 (3.5%)	
Moderately severe	25 (19%)	14 (18%)	11 (19%)	
moderate	28 (21%)	20 (26%)	8 (14%)	
Partial disability	26 (19%)	12 (16%)	14 (25%)	
Mild disability	8 (6.0%)	2 (2.6%)	6 (11%)	
Recovering	25 (19%)	18 (23%)	7 (12%)	
**QoLIBRI-6 months**				**0.8**
Impaired	34 (27%)	17 (24%)	17 (31%)	
Borderline	14 (11%)	9 (12%)	5 (9.1%)	
Normal	33 (26%)	19 (26%)	14 (25%)	
Above average	46 (36%)	27 (38%)	19 (35%)	
**GOSE-6 months**				**0.067**
Death	22 (14%)	17 (19%)	5 (8.2%)	
GR-	15 (9.9%)	9 (9.9%)	6 (9.8%)	
GR+	44 (29%)	24 (26%)	20 (33%)	
MD-	23 (15%)	13 (14%)	10 (16%)	
MD+	32 (21%)	23 (25%)	9 (15%)	
SD-	4 (2.6%)	2 (2.2%)	2 (3.3%)	
SD+	12 (7.9%)	3 (3.3%)	9 (15%)	

**Classification of TBI**				0.2
Mild	66 (41%)	36 (38%)	30 (46%)	
Moderate	55 (34%)	31 (33%)	24 (37%)	
Severe	39 (24%)	28 (29%)	11 (17%)	
[1]n (%)				
[2]Fisher's exact test; Pearson's Chi-squared test				

***GOSE:** Glasgow Outcome Scale Extended, **QoLIBRI:** Quality of Life After Brain Injury, **GR-:** Lower good recovery**, GR+:** Upper good recovery, **MD-:** Lower moderate disability, **MD+:** Upper moderate disability, **SD-:** Lower severe disability, **SD+:** Upper severe disability.

Supplementary Table S3: Correlation between injury severity and outcome at discharge and 6 months after discharge

**Characteristic**	**Mild, N = 66[1]**	**Moderate, N = 55[1]**	**Severe, N = 39[1]**	**p-value[2]**
**Disability rating scale (DRS) at discharge**				
Extreme vegetative state	0 (0%)	1 (2.0%)	4 (18%)	**0.0005**
vegetative state	0 (0%)	3 (6.0%)	0 (0%)	
Extremely severe disability	0 (0%)	4 (8.0%)	2 (9.1%)	
Severe disability	0 (0%)	5 (10%)	3 (14%)	
Moderately severe	7 (11%)	15 (30%)	3 (14%)	
Moderate	16 (26%)	7 (14%)	5 (23%)	
Partial disability	13 (21%)	9 (18%)	4 (18%)	
Mild disability	5 (8.1%)	3 (6.0%)	0 (0%)	
Recovering	21 (34%)	3 (6.0%)	1 (4.5%)	
**6-months outcome with GOSE**				
Death	1 (1.6%)	3 (6.0%)	18 (46%)	**0.0005**
Lower good recovery (GR-)	10 (16%)	4 (8.0%)	1 (2.6%)	
Upper good recover (GR+)	30 (48%)	10 (20%)	4 (10%)	
Lower moderate disability (MD-)	12 (19%)	8 (16%)	3 (7.7%)	
Upper moderate disability (MD+)	10 (16%)	13 (26%)	9 (23%)	
Lower severe disability (SD-)	0 (0%)	3 (6.0%)	1 (2.6%)	
Upper severe disability (SD+)	0 (0%)	9 (18%)	3 (7.7%)	
**6-months outcome with QoLIBRI**				
Impaired	5 (8.2%)	19 (40%)	10 (53%)	**0.0005**
Borderline	7 (11%)	6 (13%)	1 (5.3%)	

Normal	19 (31%)	9 (19%)	5 (26%)	
Above average	30 (49%)	13 (28%)	3 (16%)	
[2]Fisher's exact test				

***GOSE:** Glasgow Outcome Scale Extended, **QoLIBRI:** Quality of Life After Brain Injury, **GR-:** Lower good recovery**, GR+:** Upper good recovery, **MD-:** Lower moderate disability, **MD+:** Upper moderate disability, **SD-:** Lower severe disability, **SD+:** Upper severe disability.

Supplemental Table S4: STROBE statement

The STROBE statement — checklist of items that should be addressed in reports of cohort studies

	Item No	Recommendation	Reported on manuscript page
**Title and abstract**	1	(*a*) Indicate the study's design with a commonly used term in the title or the abstract	1
		(*b*) Provide in the abstract an informative and balanced summary of what was done and what was found	2-3
**Introduction**			
Background/rationale	2	Explain the scientific background and rationale for the investigation being reported	4-5
Objectives	3	State specific objectives, including any prespecified hypotheses	5

Methods

Study design	4	Present key elements of study design early in the paper	5-6
Setting	5	Describe the setting, locations, and relevant dates, including periods of recruitment, exposure, follow-up, and data collection	5-6
Participants	6	(*a*) Give the eligibility criteria, and the sources and methods of selection of participants. Describe methods of follow-up	5-6
		(*b*) For matched studies, give matching criteria and number of exposed and unexposed	NA
Variables	7	Clearly define all outcomes, exposures, predictors, potential confounders, and effect modifiers. Give diagnostic criteria, if applicable	6-7
Data sources/ measurement	11*	For each variable of interest, give sources of data and details of methods of assessment (measurement). Describe comparability of assessment methods if there is more than one group	7

Bias	9	Describe any efforts to address potential sources of bias	7
Study size	10	Explain how the study size was arrived at	NA (we worked with patients we received and who were willing
Quantitative variables	11	Explain how quantitative variables were handled in the analyses. If applicable, describe which groupings were chosen and why	6-7
Statistical methods	13	(*a*) Describe all statistical methods, including those used to control for confounding	8
		(*b*) Describe any methods used to examine subgroups and interactions	NA
		(*c*) Explain how missing data were addressed	15
		(*d*) If applicable, explain how loss to follow-up was addressed	We missed out 8 patients in the 6-months outcome evaluation, they were excluded from the analysis

		(*e*) Describe any sensitivity analyses	NA
**Results**			
Participants	13*	(a) Report numbers of individuals at each stage of study—eg numbers potentially eligible, examined for eligibility, confirmed eligible, included in the study, completing follow-up, and analysed	2, 8, 10
		(b) Give reasons for non-participation at each stage	NA
		(c) Consider use of a flow diagram	NA
Descriptive data	14*	(a) Give characteristics of study participants (eg demographic, clinical, social) and information on exposures and potential confounders	25-26 (Table I)
		(b) Indicate number of participants with missing data for each variable of interest	27 (Table II), 32 (Table VIIa)
		(c) Summarise follow-up time (eg, average and total amount)	NA
Outcome data	15*	Report numbers of outcome events or summary measures over time	31 (Table VI), 32 (Table VIIa),

Main results	16	(*a*) Give unadjusted estimates and, if applicable, confounder-adjusted estimates and their precision (eg, 97·5% CI's confidence interval). Make clear which confounders were adjusted for and why they were included	NA
		(*b*) Report category boundaries when continuous variables were categorized	NA
		(*c*) If relevant, consider translating estimates of relative risk into absolute risk for a meaningful time period	NA
Other analyses	17	Report other analyses done—eg analyses of subgroups and interactions, and sensitivity analyses	33-38 (Table VIIb, Supp. Table I, Supp. Table II, Supp. Table III)
**Discussion**			
Key results	18	Summarise key results with reference to study objectives	10-14
Limitations	19	Discuss limitations of the study, taking into account sources of potential bias or imprecision. Discuss both direction and magnitude of any potential bias	15

Interpretation	20	Give a cautious overall interpretation of results considering objectives, limitations, multiplicity of analyses, results from similar studies, and other relevant evidence	15-16
Generalisability	21	Discuss the generalisability (external validity) of the study results	15-16
**Other information**			
Funding	22	Give the source of funding and the role of the funders for the present study and, if applicable, for the original study on which the present article is based	NA
